# The Effect of Scandium on the Structure, Microstructure and Superconductivity of Equimolar Sc-Hf-Nb-Ta-Ti-Zr Refractory High-Entropy Alloys

**DOI:** 10.3390/ma15031122

**Published:** 2022-01-31

**Authors:** Mitja Krnel, Andreja Jelen, Stanislav Vrtnik, Jože Luzar, Darja Gačnik, Primož Koželj, Magdalena Wencka, Anton Meden, Qiang Hu, Sheng Guo, Janez Dolinšek

**Affiliations:** 1 Jožef Stefan Institute, Jamova 39, SI-1000 Ljubljana, Slovenia; mitja.krnel@ijs.si (M.K.); andreja.jelen@ijs.si (A.J.); stane.vrtnik@ijs.si (S.V.); joze.luzar@ijs.si (J.L.); darja.gacnik@ijs.si (D.G.); primoz.kozelj@ijs.si (P.K.); magdalena.wencka@ijs.si (M.W.); 2 Faculty of Mathematics and Physics, University of Ljubljana, Jadranska 19, SI-1000 Ljubljana, Slovenia; 3 Institute of Molecular Physics, Polish Academy of Sciences, Smoluchowskiego 17, PL-60-179 Poznań, Poland; 4 Faculty of Chemistry and Chemical Technology, University of Ljubljana, Večna pot 113, SI-1000 Ljubljana, Slovenia; anton.meden@fkkt.uni-lj.si; 5 Institute of Applied Physics, Jiangxi Academy of Sciences, Changdong Road 7777, Nanchang 330096, China; 6 Industrial and Materials Science, Chalmers University of Technology, SE-41296 Göteborg, Sweden; sheng.guo@chalmers.se

**Keywords:** high-entropy alloys, structure and microstructure, superconductivity

## Abstract

In this study, we investigate the scandium-containing Sc-Hf-Nb-Ta-Ti-Zr system of refractory high-entropy alloys (HEAs). Using the arc-melting method, we synthesized nine equimolar alloys (five 4-, three 5- and one 6-component), with all of them containing Sc. The alloys were characterized by XRD, electron microscopy and EDS, while superconductivity was investigated via electrical resistivity, specific heat and the Meissner effect. The results were compared to the parent Hf-Nb-Ta-Ti-Zr refractory HEAs, forming a single-phase body-centered cubic (bcc) structure and quite homogeneous microstructure. The addition of Sc produces a two-phase structure in the Sc-Hf-Nb-Ta-Ti-Zr alloys, with one phase being bcc and the other hexagonal close-packed (hcp). The hcp phase absorbs practically all Sc, whereas the Sc-poor bcc phase is identical to the bcc phase in the Hf-Nb-Ta-Ti-Zr parent system. Upon the Sc addition, the microstructure becomes very inhomogeneous. Large bcc dendrites (10–100 µm) are homogeneous in the central parts, but become a fine dispersion of sub-micron precipitates of the bcc and hcp phases close to the edges. The interdendritic regions are also a fine dispersion of the two phases. Superconductivity of the Sc-Hf-Nb-Ta-Ti-Zr alloys originates from the bcc phase fraction, which demonstrates identical superconducting parameters as the bcc Hf-Nb-Ta-Ti-Zr parent alloys, while the Sc-containing hcp phase fraction is non-superconducting.

## 1. Introduction

Refractory metals are a class of materials that demonstrate extraordinarily high resistance to heat and wear, keeping mechanical strength at high temperatures [1]. A key requirement is a high melting point. The standard definition of refractory metals includes the five elements Nb, Mo, Ta, W and Re, which all possess very high melting temperatures, $T_{m}>$ 2400 °C. A broader definition includes nine more elements (Ti, V, Cr, Zr, Ru, Rh, Hf, Os and Ir) with somewhat lower, yet still high melting temperatures. Refractory metals are chemically inert, possess high hardness at room temperature (RT) and are stable against creep deformation to high temperatures, typically T> 1500 °C (in contrast, creep in aluminum alloys starts at 200 °C). At high temperatures, they oxidize easily, but the reaction is slowed down in the bulk metal by the formation of a stable surface oxide (passivation) layer. The applications of refractory metals include tools to work metallic materials, wire filaments, casting molds and chemical reaction vessels in corrosive environments. Strong resistance against mechanical forces at high temperatures makes them potentially suitable for the application in jet engines and as tools used during forging. Shortcomings are poor low-temperature workability and extreme oxidability at high temperatures. Interaction with the environment can also significantly reduce the high-temperature creep strength, so that the application often requires protective atmosphere or coating. To overcome these shortfalls, refractory elements are frequently alloyed with other refractory elements, occasionally with an addition of a non-refractory element (such as Al). When five or more elements are alloyed in near-equimolar concentrations, crystalline random solid solutions or partially ordered ones may sometimes form, termed high-entropy alloys (HEAs) [2,3]. A well-studied refractory HEA system is Hf-Nb-Ta-Ti-Zr, which demonstrates decent RT ductility and solid solution strengthening at high temperatures [4,5,6,7,8,9,10]. This HEA is historically also the first superconducting HEA [11].

In a search for the enhancement of physical-mechanical properties of the Hf-Nb-Ta-Ti-Zr refractory HEAs, we have employed the additional alloying element, scandium (Sc). Formally classified as a rare-earth element, scandium possesses a relatively high melting temperature $T_{m}=$ 1541 °C and has a low density of 2.985 gcm^−3^. Alloying small amounts of Sc (a few %) into aluminum alloys has been demonstrated to cause a beneficial strengthening effect due to the formation of Al_3_Sc intermetallic precipitates [12], making the Sc-Al lightweight alloys for aerospace components the main application of scandium. The effect of scandium addition to multicomponent alloys has been reviewed recently [13]. Scandium possesses a strong compound-forming ability, resulting in precipitation of the Sc-containing binary and ternary intermetallic phases, so that complex multi-phase microstructures are frequently formed. Alloying 0.3–5 wt.% of Sc into 3*d* transition-metals-based HEAs Al_2_CoCrFeNi, Al_0.5_CoCrCuFeNi and AlCoCrCu_0.5_FeNi has been demonstrated to result in a powerful increase of hardness due to the precipitation-hardening effect [14]. In another work, a 3% Sc addition to the Al_2_CoCrFeNi HEA has resulted in the precipitation of the ternary Al_2_Cu_3_Sc intermetallic phase, which, in combination with high-pressure sintering, has significantly improved the hardness and thermal stability of the alloy, making it suitable for the application in extreme environments [15]. Sc has also been incorporated as one of the principal elements into equimolar AlCrCuScTi HEA [16], where it was observed that the formation and degradation of the Al_3_Sc, Al_2_Cu_3_Sc and Al_4_Cu_4_Sc intermetallic precipitates upon heating plays a crucial role in the record high value of the hardness/density ratio of this alloy, in comparison with ordinary heavy refractory HEAs. Moreover, the Sc-containing Al_20_Li_20_Mg_10_Sc_20_Ti_30_ single-phase nanocrystalline HEA has proven to be as strong as Ti, light as Al and as hard as some ceramics [17]. Sc was also used in an attempt to produce hexagonal close-packed (hcp) HEAs in the system Co-Gd-Y-Sc-Ti-Zr, which is composed only of the hcp elements [18]. It was found that single-phase hcp HEAs are not feasible within this system, but multi-phase structures are formed, with the ternary hexagonal phases ScTiZr and GdScY co-existing with one or more cubic intermetallic phases. Dual hcp structures were also found in the ScYLaTiZrHf HEA [19].

In our research, we have focused on the Sc-Hf-Nb-Ta-Ti-Zr refractory system, by synthesizing nine different 4-, 5- and 6-component alloys, targeted toward equimolar concentrations of the elements, with all of them containing the Sc element. Here we report on their structural (crystallographic), microstructural and compositional (chemical) characterization, as well as their physical properties, with the emphasis on superconductivity. To unravel the effect of the Sc addition to the Sc-Hf-Nb-Ta-Ti-Zr refractory HEAs, we compare the properties of the Sc-Hf-Nb-Ta-Ti-Zr alloys to those of the Hf-Nb-Ta-Ti-Zr parent alloys, reported before in literature [4,5,6,7,8,9,10,20,21,22,23]. The mechanical properties and corrosion resistance of the Sc-Hf-Nb-Ta-Ti-Zr alloys will be deferred to another study.

## 2. Materials Synthesis and Characterization

The properties of the individual elements constituting the investigated alloys, in the order of increasing atomic number (_21_Sc, _22_Ti, _40_Zr, _41_Nb, _72_Hf, _73_Ta), are collected in Table 1. According to the atomic radii, the elements can be grouped into two groups, one containing “big” Sc, Zr and Hf (r= 1.578–1.641 Å) and the other “small” Ti, Nb and Ta (r= 1.429–1.462 Å) elements. The high-temperature structure of all pure metals is the body-centered cubic (bcc). Nb and Ta remain bcc to RT, whereas other elements transform during cooling to hcp, which is their RT structure. The lattice parameters of the high-temperature and RT structures and the temperature of the bcc-to-hcp allotropic transition are summarized in Table 1.

The microstructure of the Sc-Hf-Nb-Ta-Ti-Zr alloys can be qualitatively predicted by considering binary-mixing enthalpies of the elemental pairs (Table 2) [24,25]. The elements Ti, Zr, Nb, Hf and Ta mix well, as the binary-mixing enthalpies of any pair are close to zero (do not exceed 4 kJ mol^−1^). Scandium mixes relatively well with the Ti, Zr and Hf, but experiences strong repulsion to Nb ($\Delta H_{mix}^{ScNb}=$ 18 kJ mol^−1^) and Ta ($\Delta H_{mix}^{ScTa}=$ 16 kJ mol^−1^). According to this criterion, more than one phase can be expected in the alloys, where the Ta- and Nb-rich phases will be poor in Sc and vice versa. Non-random mixing of the elements can also be expected on the basis of different atomic radii, where clustering of the elements with similar radii minimizes the lattice deformation (strain) energy that contributes to the total mixing enthalpy of the alloy.

We have synthesized nine different alloys, of which there were five 4-component, three 5-component and one 6-component. All alloys were targeted towards equimolar concentrations of the elements and Sc was a common element to all of them. The alloys are listed in Table 3. By naming the alloys, Sc is always put on the first place, whereas other elements are arranged in alphabetical order. For easier “bookkeeping”, the alloys were also assigned consecutive numbers #1–#9. The five 4-component alloys are #1-ScHfNbTi, #2-ScHfNbZr, #3-ScHfTaTi, #4-ScNbTiZr and #5-ScTaTiZr, and the three 5-component alloys are #6-ScHfNbTaTi, #7-ScHfNbTaZr and #8-ScNbTaTiZr, whereas the 6-component alloy is #9-ScHfNbTaTiZr. The method of preparation was arc melting in an Ar atmosphere, where each ingot was remelted several times and cooled down naturally, so that the materials were in an as-cast state. The determination of melting temperatures of refractory alloys is a known problem, because most commercial differential scanning calorimeters (DSC) cannot reach high enough temperatures. Our alloys were heated up to 1600 °C in the DSC experiments (not shown) and no melting of the alloys could be detected up to that temperature, consistent with their refractory character. The samples for measurements were cut from the middle parts of the ingots, where the microstructure was more homogeneous than at the edges. Structural (crystallographic), microstructural and chemical characterization was performed by X-ray diffraction (XRD), scanning electron microscopy backscattered-electron (SEM BSE) imaging, energy-dispersive X-ray spectroscopy (EDS) and elemental mapping. The experimental details are described in the Methods Section.

### 2.1. X-ray Diffraction

**Figure 1:**
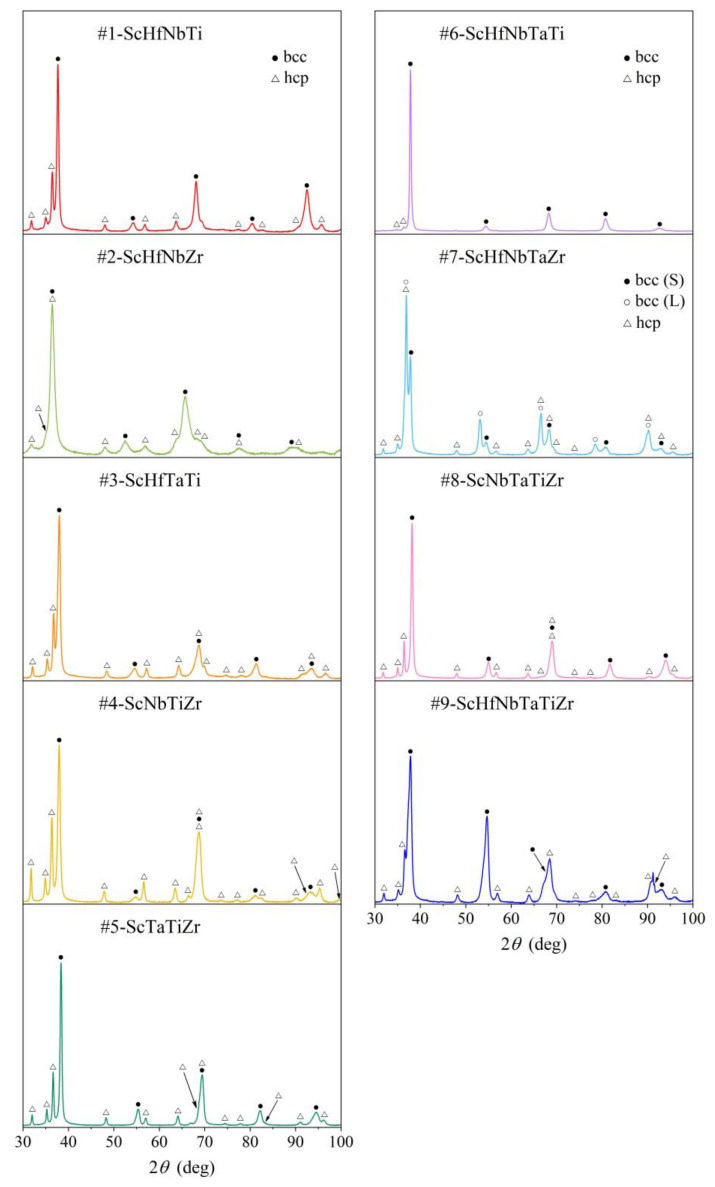
XRD patterns of the investigated Sc-Hf-Nb-Ta-Ti-Zr alloys.

XRD patterns of the investigated alloys are presented in Figure 1. In all cases, a two-phase structure was found, with one phase being bcc (space group *Im*$\bar{3}$*m*, No. 229), the other hcp (space group *P*6_3_/*mmc*, No. 194). The bcc and hcp lattice parameters are summarized in Table 3. The bcc parameter of most alloys is in the range a= 3.32–3.37 Å, with two exceptions. In the #2-ScHfNbZr alloy, it is a bit larger and amounts to a= 3.47 Å, whereas in the #7-ScHfNbTaZr alloy, two bcc phases could be discerned, one with a smaller lattice parameter $a_{S}=$ 3.37 Å (classifying in the abovementioned range of most of the alloys) and the other with a larger $a_{L}=$ 3.45 Å (very similar to the one in the #2-ScHfNbZr alloy). For the hcp phase, the lattice parameters are in the ranges a= 3.23–3.26 Å and c= 5.09–5.15 Å. Molar fractions of the bcc and hcp phases, as determined from the XRD peak intensities, vary between the alloys (Table 3). The bcc phase is the majority phase in all alloys, with its molar fraction ranging between 60% and 80% (the estimated error is ±5%).

### 2.2. The Microstructure

**Figure 2:**
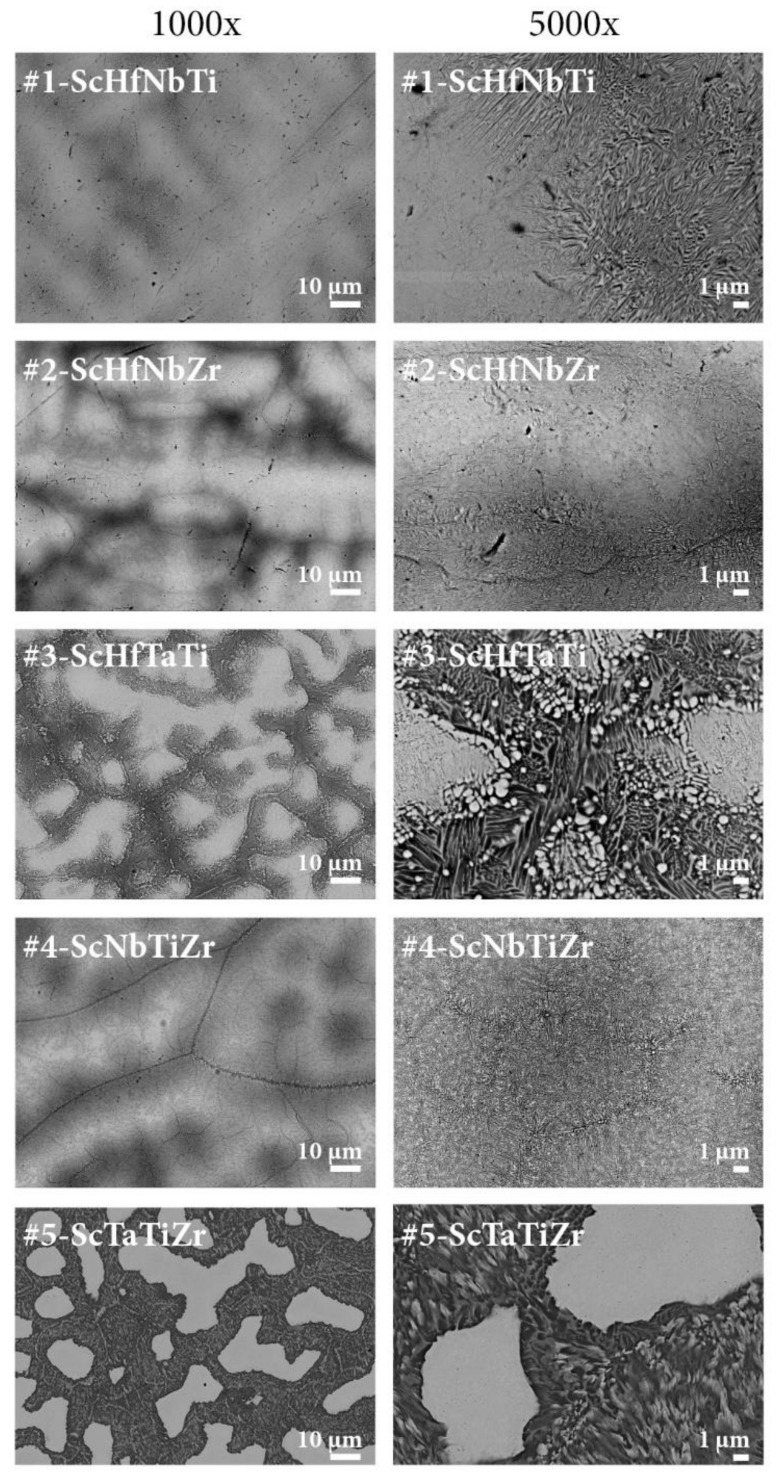

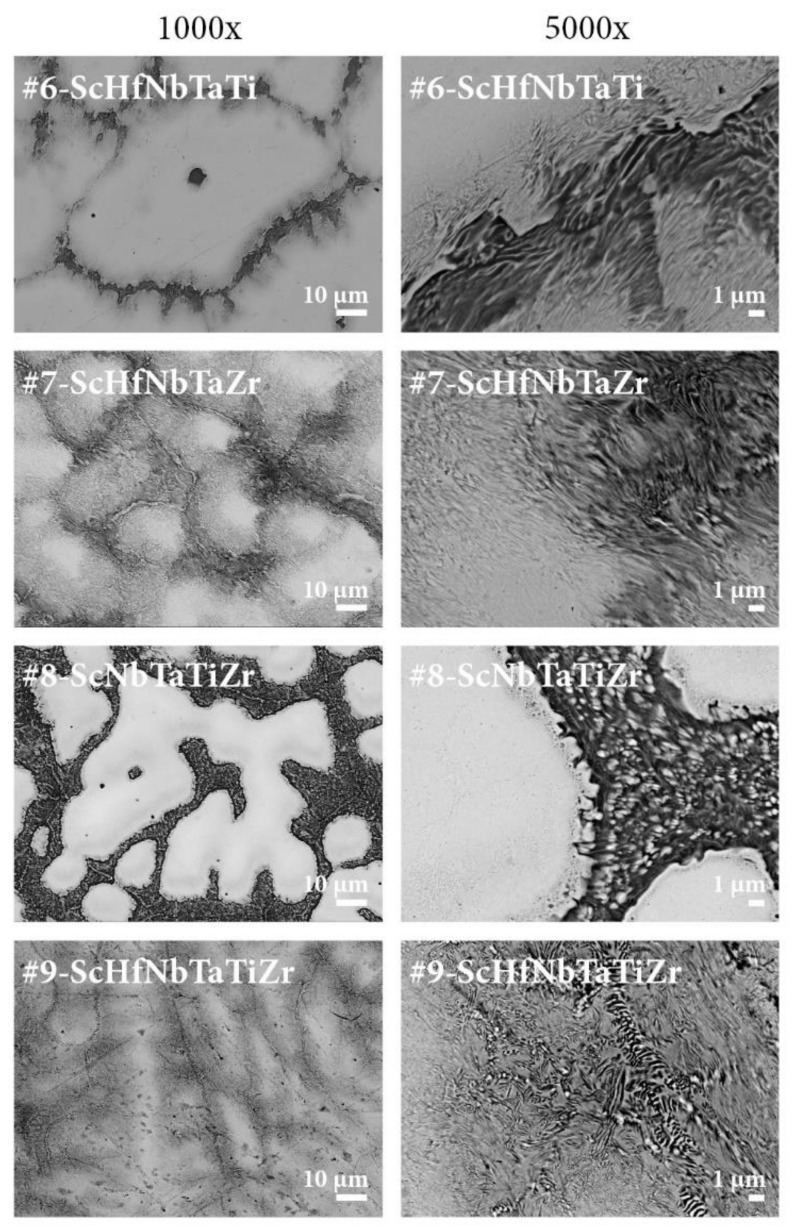
SEM BSE images of the Sc-Hf-Nb-Ta-Ti-Zr alloys #1–#9 at two magnifications, 1000× (**left** column) and 5000× (**right** column).

SEM BSE images of the alloys are presented in Figure 2 at two magnifications, 1000× (left column) and 5000× (right column). In all cases, the microstructure is very inhomogeneous, containing large dendrites (sometimes in the shape of “islands”) of up to several 100-µm dimension that appear bright on the images due to enrichment in heavier elements, and separated by darker interdendritic regions that are enriched in lighter elements. The phase-separated microstructure is present in all alloys, but is more pronounced in the six alloys that contain the tantalum element. The dendrites are more or less pure-phase (bright) in the middle parts, forming large single-phase regions of several 10 to 100 µm dimensions. Towards the edges, a fine dispersion of much smaller precipitates of the two phases (bright and dark), mostly of 10–100 nm dimensions, is evident on the sub-micron scale. The dark interdendritic regions, enriched in lighter elements, are even more inhomogeneous. There are no large (µm-size) regions of either of the two phases, but the microstructure is more mottled, containing finely intermixed bright and dark precipitates of the two phases (of spherical-like or elongated shape), again of 10–100 nm dimensions. The dark precipitates occupy considerably larger fraction of the interdendritic regions than the bright ones. The interdendritic regions can be viewed as eutectic mixtures.

### 2.3. EDS Chemical Analysis

In SEM EDS, the interaction volume of the beam electrons with the atoms of the sample is typically of a few µm cross-dimension. Consequently, the chemical composition of the two phases discerned by BSE imaging could be reliably determined only for the bright phase in the large homogeneous (central) parts of the dendrites/islands. Close to the edges and in the entire interdendritic regions, the finely dispersed bright and dark precipitates of sub-micron dimensions prevented the accurate analysis of chemical composition of the individual phases, but only an average composition could be determined. As there are no uniform dark regions of about 10-µm dimension present in the microstructure of any of the alloys, we were unable to determine the exact chemical composition of the dark phase. The EDS results are summarized in Table 3, where one should keep in mind that the reported compositions of the dark phase are an average of the elements present in the interdendritic eutectic mixtures.

**Figure 3:**
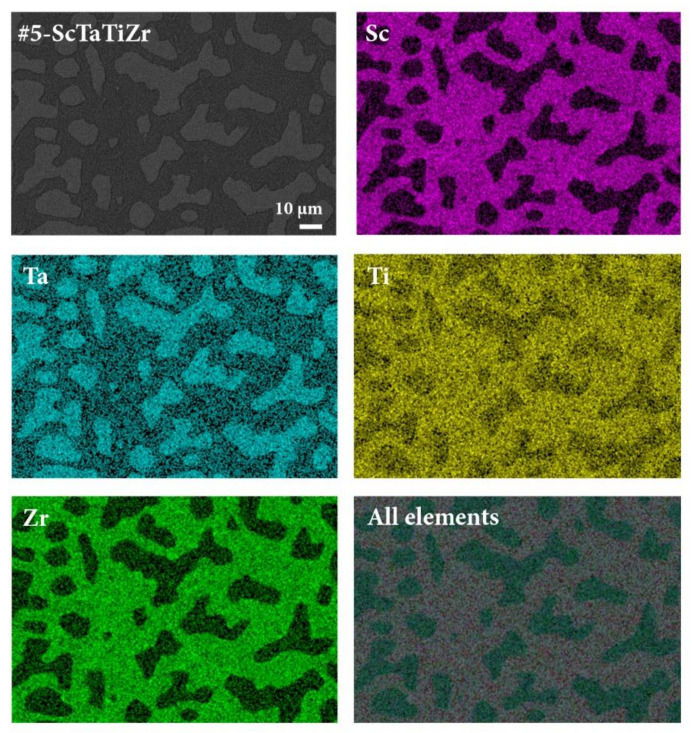
EDS elemental maps of the #5-ScTaTiZr alloy. The elemental maps of other eight alloys are presented in the Supplementary Materials.

The EDS elemental maps of the 4-component #5-ScTaTiZr alloy are shown in Figure 3, whereas the elemental maps of other eight alloys are collected in the Supplementary Materials. The bright islands of composition Sc_4_Ta_64_Ti_22_Zr_10_ (in at.%) are strongly enriched in Ta and include a significant amount of Ti, but are depleted in Zr and contain almost no Sc (the detected small amount of 4 at.% Sc may still be dispersed in this phase, but it is also possible that the Sc EDS signal is collected from the environment, e.g., below the islands). The interdendritic region of composition Sc_32_Ta_10_Ti_26_Zr_32_ is Sc- and Zr-rich and also contains a lot of Ti, but is depleted in Ta. By also analyzing the EDS elemental maps of other alloys, we found that the following composition pattern is commonly appearing in all alloys: the bright dendrites are Ta- and Nb-rich, but poor in Sc and Zr, whereas the opposite holds true for the dark interdendritic regions, which are Sc- and Zr-rich, but Ta- and Nb-poor. The dark phase absorbs practically all Sc. Hf and Ti are quite uniformly distributed over the two phases. The fact that Sc “likes” Zr, but strongly repels Ta and Nb, whereas Hf and Ti mix well with all elements, is in agreement with the qualitative predictions based on the binary mixing enthalpies from Table 2. The considerable amount of Ta detected in the interdendritic regions of some alloys (up to 15 at.%) does not prove its inclusion into the dark phase, but rather reflects fine dispersion of the dark and bright sub-micron precipitates that are simultaneously hit by the electron beam in the EDS experiment.

By combining the results of the XRD, SEM BSE and EDS analyses, it is straightforward to associate the bright regions with the bcc phase, while the dark regions are hcp. The bcc phase is Ta- and Nb-rich, but Sc- and Zr-poor and the opposite holds for the hcp phase. Scandium is responsible for the formation of the hcp phase and more or less all Sc is absorbed in this phase. Minor inclusion of Sc into the bcc phase (up to a few at.%) cannot be ruled out.

## 3. Superconductivity in the Sc-Hf-Nb-Ta-Ti-Zr Alloys

The Sc-Hf-Nb-Ta-Ti-Zr system comprises five superconducting (SC) elements Hf, Nb, Ta, Ti and Zr, all belonging to the class of low-temperature superconductors, whereas Sc is non-superconducting at ambient pressure. The SC transition temperatures $T_{C}$ of pure metals are collected in Table 4, ranging from the highest $T_{C}^{Nb}=$ 9.20 K to the lowest $T_{C}^{Hf}=$ 0.12 K. We have studied the SC transition via electrical resistivity, specific heat and the Meissner effect. The experimental details are described in the Methods Section.

### 3.1. Electrical Resistivity

**Figure 4:**
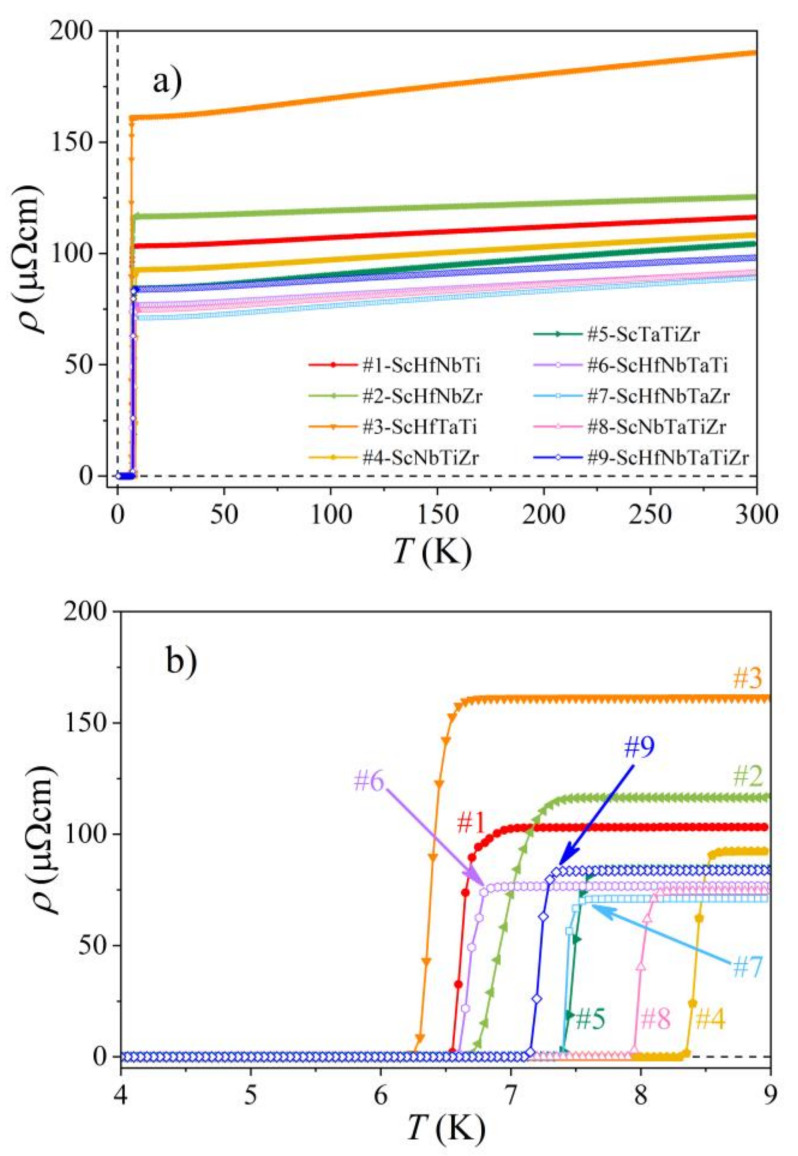
(**a**) Electrical resistivity of the Sc-Hf-Nb-Ta-Ti-Zr alloys in the temperature range 0.35–300 K. (**b**) Resistivities on an expanded temperature scale below 9 K.

Temperature-dependent electrical resistivity $\rho \left(T\right)$ was measured between 0.35 and 300 K and the results are presented in Figure 4a. The normal-state resistivities of all alloys exhibit very weak temperature dependence with a positive temperature coefficient and their RT values are high (in the range 90–190 µΩcm), typical of disordered metals and alloys. This is an indication of prevalent elastic scattering of conduction electrons by quenched defects (lattice distortions, grain boundaries and chemical disorder) within the normal state. A transition to the zero-resistance (superconducting) state is observed in all alloys at temperatures below 10 K. The transition temperature determined from the electrical resistivity is denoted in the following as $T_{C}^{\rho }$. It is defined in the standard way as the temperature where the resistivity reaches half of the normal-state value just above the transition. The transition temperatures assume values in a relatively narrow interval $T_{C}^{\rho }=$ 6.4–8.4 K, where $T_{C}^{\rho }=$ 8.4 K of the #4-ScNbTiZr is the highest and $T_{C}^{\rho }=$ 6.4 K of the #3-ScHfTaTi is the lowest. In Figure 4b, the resistivities are demonstrated on an expanded temperature scale below 9 K, where it is evident that the transition region in some alloys is narrower and in the others broader, reflecting different degrees of structural and chemical disorder in the samples. The $T_{C}^{\rho }$ values are collected in Table 4.

**Figure 5:**
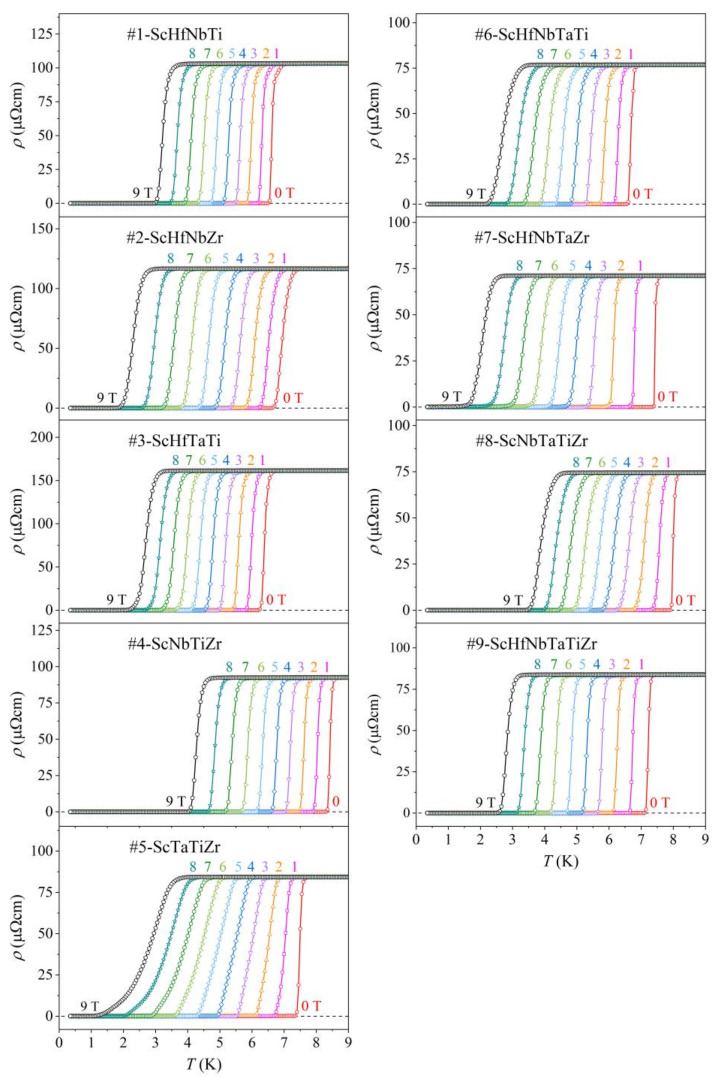
Magnetic-field-dependent electrical resistivity in the region of the superconducting transition for fields 0–9 T.

**Figure 6:**
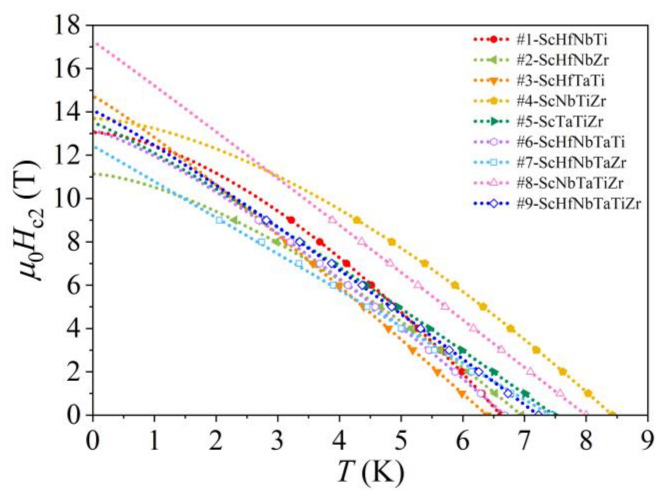
Temperature-dependent upper critical field $H_{c2}$ of the Sc-Hf-Nb-Ta-Ti-Zr alloys.

Magnetic-field dependence of the resistivity at the SC transition was studied in the magnetic field range, B= 0–9 T. The field-dependent resistivity curves in the transition region are shown in Figure 5. As expected, the SC transition shifts with the field to lower temperatures. The $T_{C}^{\rho }\left(B\right)$ values were used to determine the temperature dependence of the upper critical field $H_{c2}$ of a type II superconductor. The $H_{c2}\left(T\right)$ curves are presented in Figure 6. The T→0 extrapolated values $H_{c2}\left(0\right)$ of all alloys were determined from the empirical fits $H_{c2}\left(T\right)\;=H_{c2}\left(0\right)\left[1-{\left(T/T_{C}^{\rho }\right)}^{\beta }\right]$, yielding $\mu_{0}H_{c2}\left(0\right)$ values in the range 11.1–17.3 T (these values, together with the exponents, β, are collected in Table 4).

### 3.2. Specific Heat

Low-temperature specific heat C of inhomogeneous (“dirty”) superconductors gives valuable information on the details of the SC transition, related to the structural and chemical disorder. At temperatures below about 10 K, the total specific heat of the normal state can be written as $C_{n}=\gamma T+\alpha T^{3}$, where γ and α are the electronic and lattice specific heat coefficients, respectively. The specific heat of the SC state is $C_{s}=C_{es}+\alpha T^{3}$, where $C_{es}$ is the specific heat of the superconducting-electron system. At the SC transition temperature $T_{C}$ (which is usually a bit lower than the $T_{C}^{\rho }$ determined from the resistivity), the specific heat exhibits a discontinuity (a jump) that equals the difference in the electronic specific heats of the SC and normal states due to the formation of Cooper pairs [26],

(1) $$\Delta C\left(T_{C}\right)\;=C_{s}\left(T_{C}\right)\;-\;C_{n}\left(T_{C}\right)\;=C_{es}\left(T_{C}\right)\;-\;\gamma T_{C}.\;$$

In homogeneous superconductors, the slope of the discontinuity on the high-temperature (normal-state) side is infinitely steep, reflecting the fact that the entire material becomes superconducting at the same temperature, i.e., $T_{C}$ is the same for all parts of the sample. In chemically and structurally disordered superconductors, different parts of the material become SC at slightly different temperatures, so that $T_{C}$ is smeared (distributed) and the slope of the discontinuity on the high-temperature side is gentle (less steep). The sharpness of the discontinuity at $T_{C}$ is a reliable indication of the material’s (in)homogeneity.

Specific heat can also be used to determine the fraction of the SC phase in the T→0 limit for samples that are not SC in the entirety of their volumes. Such cases are a mixture of SC and normal regions in inhomogeneous materials and a vortex state in type II superconductors. Denoting the fraction of the SC phase by x (with 0≤x≤1), whereas 1−x is the fraction of the normal phase, the specific heat of a “mixture” can be written as [26]:

(2) $$C=xC_{es}+\;\left(1-x\right)\gamma T+\alpha T^{3}.$$

The analysis is conveniently performed in the C/T vs. $T^{2}$ presentation. In the T→0 limit, $C_{es}$ vanishes exponentially and so does $C_{es}/T$, yielding the zero-temperature value ${\left(C/T\right)}_{T=0}=\left(1-x\right)\gamma$. As γ can be determined independently from the specific heat measurement in a magnetic field $H>H_{c2}$ (where the entire sample is in the normal state), the extrapolated experimental zero-field ${\left(C/T\right)}_{T=0}$ value enables the determination of the SC fraction x. In addition, the $C/T=\gamma +\alpha T^{2}$ analysis of the normal-state specific heat allows for the determination of α and consequently the Debye temperature $\theta_{D}={\left(12\pi^{4}R/5\alpha \right)}^{1/3}$, where R is the gas constant.

**Figure 7:**
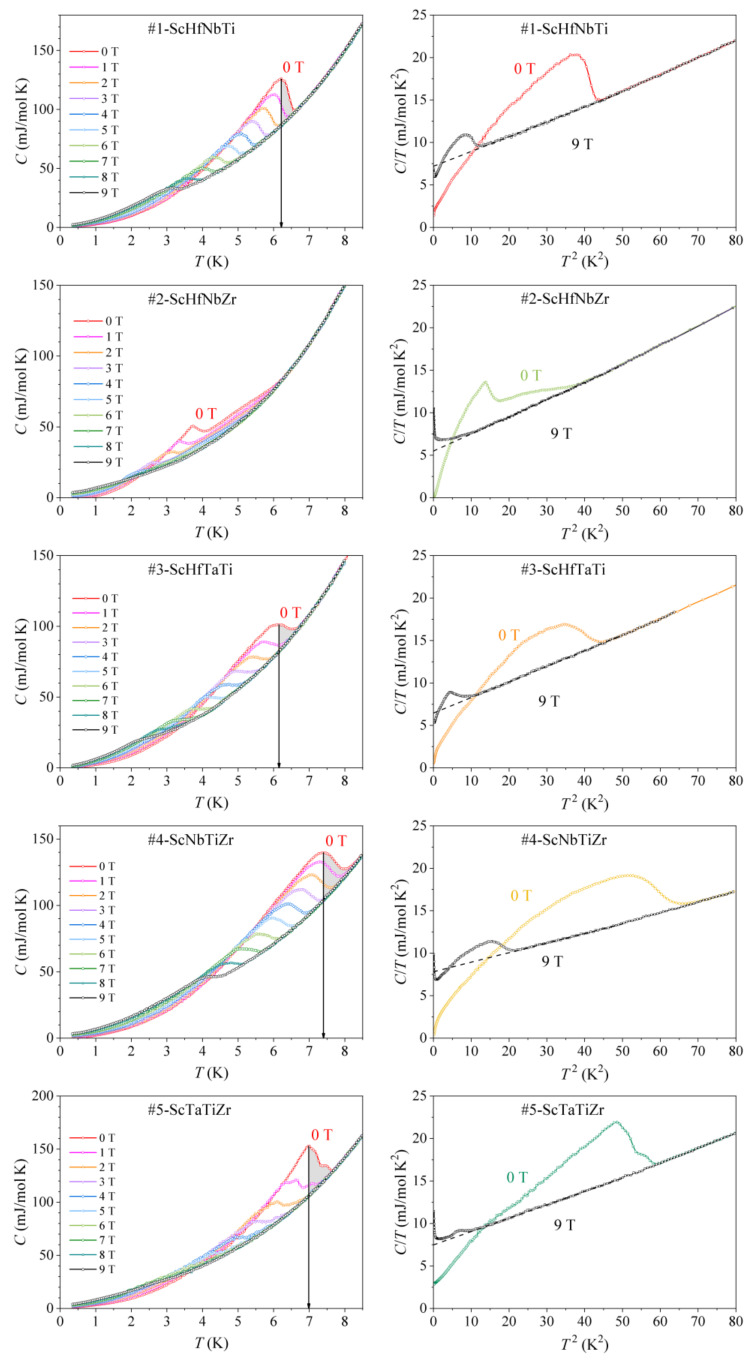

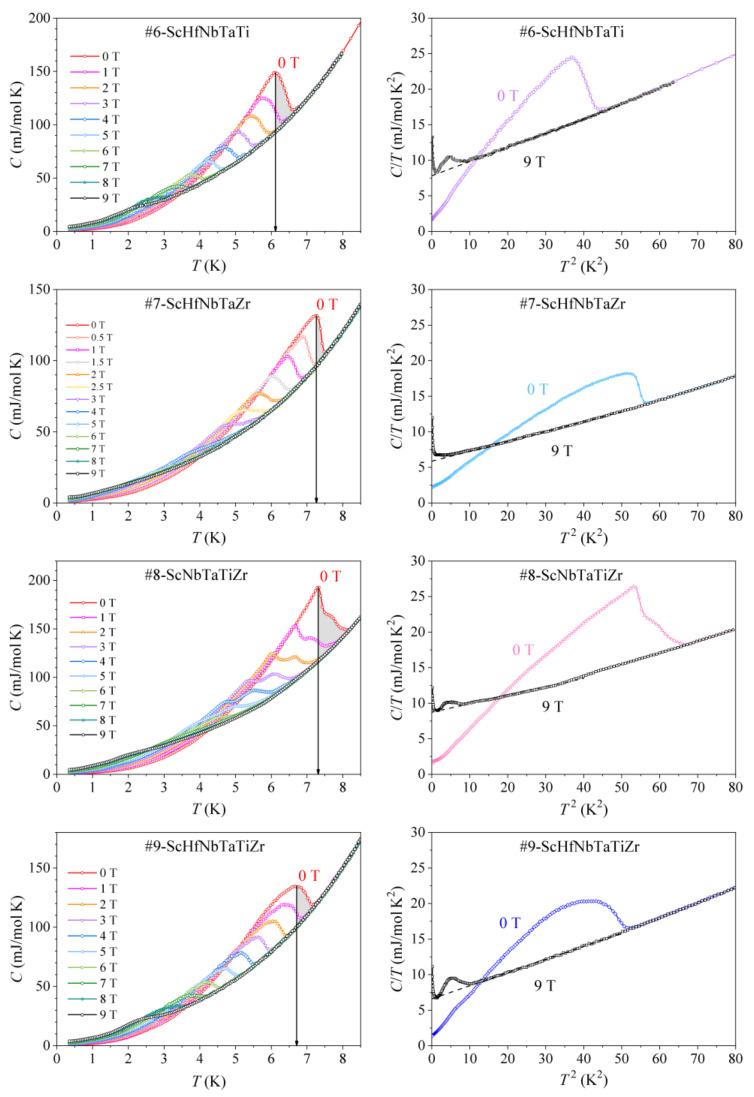
Low-temperature specific heat $C\left(T\right)$ of the Sc-Hf-Nb-Ta-Ti-Zr alloys at selected magnetic fields in the range 0–9 T (**left** column). Vertical arrows denote the zero-field SC transition temperature $T_{C}$ (except for the #2-ScHfNbZr alloy). In the **right** column, the zero-field and the 9-T specific heat is shown in a C/T vs. $T^{2}$ plot. Dashed lines are fits of the normal-state specific heat with the expression $C/T=\gamma +\alpha T^{2}$.

The low-temperature specific heat of all alloys in the temperature range 0.35–8.5 K, determined in the magnetic fields B= 0–9 T, is shown in the left column of Figure 7 in a C vs. T plot. The SC transition temperature $T_{C}$ is defined as the temperature where the discontinuity exhibits the peak. Some alloys show a single, broad discontinuity at $T_{C}$, whereas others show a double-peak-type discontinuity. The #2-ScHfNbZr alloy is somewhat special, as the double-peak-type discontinuity is considerably broader than in the other alloys. In all alloys, the discontinuity decreases in intensity and shifts to lower fields with increasing B, reflecting the temperature dependence of the upper critical field $H_{c2}\left(T\right)$. In all graphs (except that of the #2-ScHfNbZr alloy), the zero-field $T_{C}$ has been marked by a vertical arrow, pointing to the peak of the discontinuity measured in B=0. The inhomogeneity of the material is then conveniently visualized by shading the area under the discontinuity on the high-temperature side of this vertical line. Large shaded areas are fingerprints of high chemical and structural inhomogeneity of the materials.

The parameters γ, $\theta_{D}$ and the molar SC fraction x of each alloy were determined from the C/T vs. $T^{2}$ plots of the specific heat measured in magnetic fields 0 and 9 T, shown in the right column of Figure 7. The 9-T data of all alloys indicate that the field of 9 T (the highest field available in our experiments) is close, but still below the upper critical field $\mu_{0}H_{c2}\left(0\right)$ (otherwise the 9-T data should fall on a straight line down to T= 0), but the data are clear enough that a reliable normal-state fit $C/T=\gamma +\alpha T^{2}$ could be performed (dashed lines in the right column of Figure 7). The so determined γ and $\theta_{D}$ values are average values over the bcc and hcp phases in the normal state and are collected in Table 4. The γ values are distributed in the range γ= 5.50–8.74 mJ mol^−1^ K^−2^, with the alloy #8-ScNbTaTiZr showing the highest and the alloy #2-ScHfNbZr the lowest γ value. The Debye temperatures are in the range $\theta_{D}=$ 213–259 K. These values are to be contrasted with the γ and $\theta_{D}$ values of pure metals, which are also given in Table 4. The γ values of the Sc-Hf-Nb-Ta-Ti-Zr alloys fall in the range of the γ values of pure metals and the $\theta_{D}$ values are also comparable to those of pure metals.

The SC-phase molar fraction x of each alloy at T→ 0 is presented in the last column of Table 4. All alloys are a mixture of a SC and a normal (non-SC) phase, with x in the range 61–86% (the estimated experimental precision is ±5%).

### 3.3. Meissner Effect

The Meissner effect, where the magnetic flux is expelled out of the superconductor below the critical temperature $T_{C}$, is another manifestation of superconductivity. In type II superconductors, the flux lines are entirely expelled out of the material for magnetic fields below the lower critical field $H_{c1}$ and the volume magnetic susceptibility χ=M/H consequently assumes the ideal diamagnetic value χ= −1 (in SI units). Upon sweeping the magnetic field from zero in the magnetization vs. the magnetic field experiment, the $M\left(H\right)$ curve is linear with a negative slope −1 for the fields $0\leq H\leq H_{c1}$. At $H_{c1}$, it exhibits a minimum and then increases until the magnetic susceptibility reaches its normal-state value. Plotting the temperature of the $M\left(H\right)$ minimum as a function of the magnetic field enables the determination of the lower critical field $H_{c1}\left(T\right)$.

**Figure 8:**
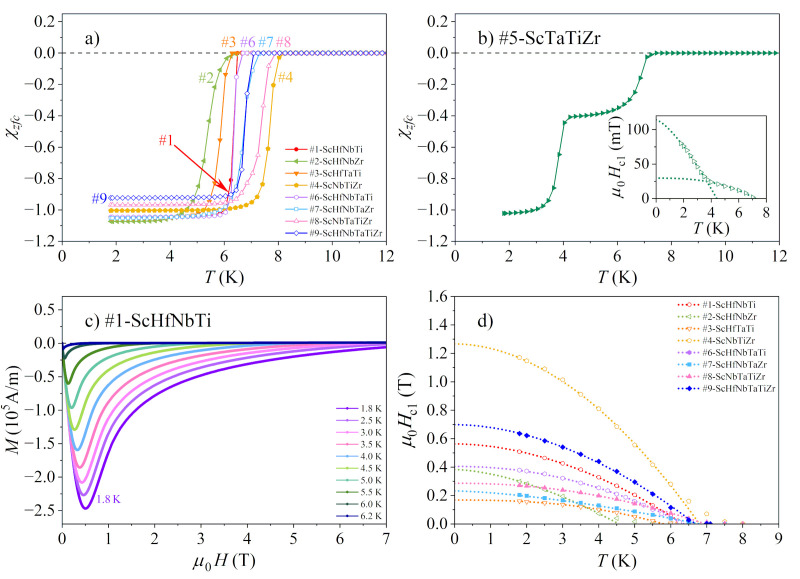
(**a**) Low-temperature magnetic susceptibility $\chi \left(T\right)$ of the Sc-Hf-Nb-Ta-Ti-Zr alloys (except #5-ScTaTiZr), measured for the zero-field-cooled protocol in a magnetic field $\mu_{0}H_{zfc}=$ 5 mT. (**b**) Susceptibility of the #5-ScTaTiZr alloy. (**c**) $M\left(H\right)$ curves of the 1#-ScHfNbTi alloy in the field range 0–9 T at different temperatures within the SC phase (this set of curves is representative of all alloys). (**d**) Temperature-dependent lower critical field $H_{c1}\left(T\right)$ of all alloys except #5-ScTaTiZr. The $H_{c1}\left(T\right)$ curve of the alloy #5-ScTaTiZr is shown in the inset of panel (**b**).

The magnetic susceptibility $\chi \left(T\right)$ of the investigated alloys (except the one of the #5-ScTaTiZr, which will be presented separately), measured for the zero-field-cooled protocol in a weak magnetic field $\mu_{0}H_{zfc}=$ 5 mT, is shown in Figure 8a in the temperature range of the SC transition. The Meissner effect is clearly evident for all alloys. The steepness of the χ drop at the SC transition varies between the alloys, reflecting different degrees of the samples’ inhomogeneity (for more homogeneous samples, the drop is more abrupt). The $\chi \left(T\right)$ of the #5-ScTaTiZr alloy is shown in Figure 8b, where two successive steps are evident, one at about 7 K and the other at 4 K. The temperature of the upper step (7 K) matches the $T_{C}=$ 6.8 K well, determined from the specific heat maximum. There are obviously two successive SC transitions in this alloy, though the lower one (4 K) is not noticeable in the specific heat curve. The two transitions observable in the $\chi \left(T\right)$ can be related to the highly inhomogeneous microstructure of this alloy, which consists of the well-formed “islands” of the bcc phase and the matrix that is a fine dispersion of the bcc and hcp sub-micron precipitates (Figure 2). It is likely that the islands turn SC at the upper step temperature, whereas the bcc precipitates in the matrix become SC at the lower step temperature due to a slightly different chemical composition.

The lower critical field $H_{c1}$ was determined from the $M\left(H\right)$ curves, measured in the field range 0–9 T at different temperatures within the SC phase. A representative set of the $M\left(H\right)$ curves is shown in Figure 8c for the 1#-ScHfNbTi alloy. The $H_{c1}\left(T\right)$ curves of all alloys except #5-ScTaTiZr are presented in Figure 8d. The lower critical field values $H_{c1}\left(0\right)$ were determined by the same type of empirical fit as the upper critical field, this time with the exponent $\beta_{1}$. The lower critical field values $\mu_{0}H_{c1}\left(0\right)$ are in the range 0.17–1.27 T. The $H_{c1}\left(T\right)$ curve of the alloy #5-ScTaTiZr, which shows a two-step transition in the $\chi \left(T\right)$ curve, is shown in the inset of Figure 8b. It is evident that two lower critical fields can be defined, $\mu_{0}H_{c1}^{\left(1\right)}=$ 0.03 T and $\mu_{0}H_{c1}^{\left(2\right)}=$ 0.11 T. All $\mu_{0}H_{c1}\left(0\right)$ values are also collected in Table 4.

## 4. Discussion

According to the commonly used criterion for the classification of ideal solid solutions based on their entropy of mixing, $\Delta S_{mix}=-R\sum_{i=1}^{N}c_{i}lnc_{i}$ (where N is the number of components and $c_{i}$ is the concentration of component i), 4-component solid solutions with $1R<\Delta S_{mix}<1.5R\;$ belong to the class of medium-entropy alloys (MEAs), whereas solid solutions with $\Delta S_{mix}>1.5R$, obtained for N≥5 are HEAs [2,3,27,28,29]. Our investigated Sc-Hf-Nb-Ta-Ti-Zr two-phase alloys are far from ideal solid solutions, raising the question of whether this type of classification is meaningful or their classification as “compositionally complex alloys—CCAs” is more appropriate. It is, however, common practice in literature to retain the classification of such inhomogeneous alloys in terms of MEAs and HEAs, meaning we keep this denotation. Accordingly, the five 4-component alloys from the investigated Sc-Hf-Nb-Ta-Ti-Zr system are MEAs, whereas the three 5-component and one 6-componet alloys are HEAs.

By considering the effect of Sc on the crystal structure and microstructure of the senary Sc-Hf-Nb-Ta-Ti-Zr system, it is instructive to review first the structural and microstructural features of the quinary Hf-Nb-Ta-Ti-Zr parent system, which has been studied extensively in literature [4,5,6,7,8,9,10,20]. The as-cast Hf-Nb-Ta-Ti-Zr HEAs of various chemical compositions are fully single-phase, crystallizing in the bcc structure with the lattice parameter in the range a= 3.36–3.37 Å. For some compositions, two bcc phases were detected in the same material, one with a smaller parameter $a_{S}\approx$ 3.36 Å and the other with a larger $a_{L}\approx$ 3.40–3.48 Å. The situation is completely analogous to the bcc phase in the Sc-Hf-Nb-Ta-Ti-Zr alloys (see Table 3), indicating that the bcc phase fraction in the Sc-Hf-Nb-Ta-Ti-Zr could be the same as the bcc phase in the Hf-Nb-Ta-Ti-Zr parent system. This also suggests that the bcc phase in the Sc-Hf-Nb-Ta-Ti-Zr alloys likely does not contain any Sc, i.e., all Sc binds into the hcp phase. According to the EDS analysis summarized in Table 3, a minor amount of Sc (up to about 4%) within the bcc phase cannot be excluded, but its possible presence does not noticeably influence the crystallographic parameters and the microstructure of the bcc phase.

The microstructure of the as-cast Hf-Nb-Ta-Ti-Zr parent system contains dendrites slightly enriched in Nb and Ta, separated by interdendritic regions enriched in Hf, Ti and Zr [4,5,6,7,8,9,10]. However, all five elements are still quite uniformly dispersed over the 50–100 µm distance, with the variation of the elemental concentrations by up to 6% for Ta, 4% for Zr and 2% for other elements. The (in)homogeneity of the elemental distribution depends on the cooling rate, where faster cooling rates yield a more homogeneous distribution. Thermal annealing largely dissolves the dendrite arms and the variations in the elemental concentrations become smaller—below 2% for all elements after one day. An interesting evolution upon thermal annealing was also observed in the nanostructure on the scale of 10–100 nm [4]. While the as-cast material shows homogeneous distribution of the elements on the nanoscale, thermal annealing results in a formation of planar short-range atomic clusters enriched in Hf and Zr along the ⟨100⟩ crystallographic directions, forming a connected three-dimensional (3D) grid after the annealing time of about one day. The spacing between the short-range clusters is in the range 7–15 nm. As the binary mixing enthalpies of any pair of the elements Hf, Nb, Ta, Ti and Zr are very close to zero (Table 2), which is preferential condition for random mixing, clustering of the “big” Hf and Zr atoms away from the “small” Nb, Ta and Ti can be understood as the endeavor of the alloy to minimize the lattice strain energy by grouping the elements of similar atomic radii. To summarize, the Hf-Nb-Ta-Ti-Zr parent HEAs are single-phase bcc materials, with close-to-random mixing of the elements on the 1–100 µm scale that results in quite homogeneous microstructures.

The addition of scandium has a detrimental effect on both the single-phase character of the Sc-Hf-Nb-Ta-Ti-Zr alloys and the homogeneity of their microstructure. Scandium is responsible for the appearance of the hcp phase fraction, which absorbs more or less all Sc, but is poor in Ta and Nb. The resulting two-phase structure is highly inhomogeneous. Large Ta- and Nb-rich, but Sc-poor bcc dendrites (or islands) of several 10 to 100 µm cross dimension are formed, which are quite homogeneous in the interior, but become a fine dispersion of sub-micron precipitates of the bcc and hcp phases close to the edges. A similar fine dispersion of the sub-micron bcc and hcp precipitates (where the latter are in majority) is also characteristic of the interdendritic regions, which do not contain any large, µm-size hcp regions.

**Figure 9:**
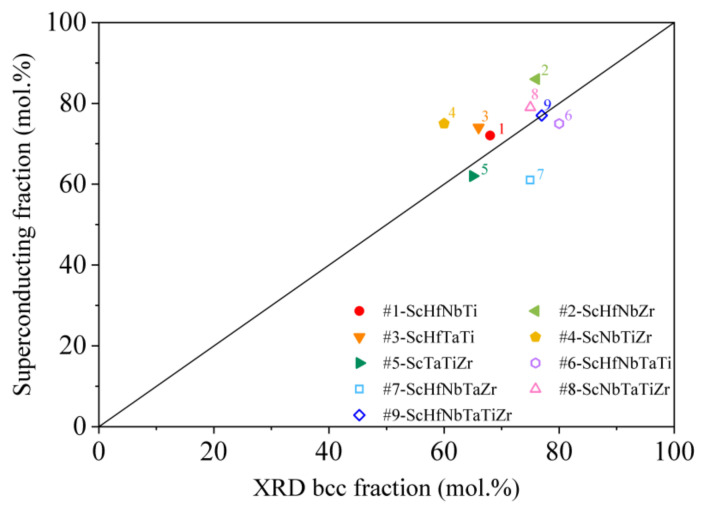
The superconducting molar fraction x versus the XRD-determined molar fraction of the bcc phase in the Sc-Hf-Nb-Ta-Ti-Zr alloys.

Superconductivity of the Sc-Hf-Nb-Ta-Ti-Zr system is closely related to the parent Hf-Nb-Ta-Ti-Zr system. For the latter, it was demonstrated that the superconductivity is very robust, appearing for practically any composition of the elements (within the range 8–35 at.% for each element) and the SC phase fraction at T→0 is 100%, i.e., the alloys are SC in the entirety of their volumes [11,20,21,22,23]. The SC transition temperatures of the Hf-Nb-Ta-Ti-Zr parent alloys of various compositions are in the range $T_{C}\approx$ 6–8 K, which is the same range as the $T_{C}$s of the Sc-Hf-Nb-Ta-Ti-Zr alloys. The upper critical fields of the parent alloys and the Sc-containing ones are also in the same range. As the Sc-Hf-Nb-Ta-Ti-Zr two-phase alloys are not fully SC at T→0, this raises the question whether only the bcc (Sc-poor) phase fraction of Sc-Hf-Nb-Ta-Ti-Zr participates in the superconductivity, whereas the Sc-rich hcp phase fraction does not turn superconducting. If so, this would explain why the superconducting parameters of the Sc-Hf-Nb-Ta-Ti-Zr alloys are practically equal to those of the bcc Hf-Nb-Ta-Ti-Zr parent alloys. The answer is obtained by plotting the superconducting molar fraction x of the Sc-Hf-Nb-Ta-Ti-Zr alloys from Table 4 versus the XRD-determined molar fraction of the bcc phase from Table 3. The graph is shown in Figure 9, where a clear linear correlation is observed, where larger bcc phase fraction correlates with larger x. This gives strong support to the hypothesis that only the Sc-poor bcc phase fraction is superconducting, whereas the Sc-rich hcp phase fraction is not. The non-superconducting element scandium obviously suppresses superconductivity of the hcp phase fraction.

There is thus a strong indication that the superconductivity of the Sc-Hf-Nb-Ta-Ti-Zr alloys originates from the bcc phase fraction, which shows practically identical crystallographic and superconducting parameters as the bcc Hf-Nb-Ta-Ti-Zr parent alloys. The latter are well documented in literature [11,20,21,22,23], so that there is no need for further analysis of the properties of the SC phase in the investigated Sc-Hf-Nb-Ta-Ti-Zr alloys. The phase belongs to the class of type II superconductors and is close to a Bardeen–Cooper–Schrieffer (BCS)-type phonon-mediated superconductor in the weak electron-phonon coupling limit [30]. The enormous chemical and structural disorder allows its description in terms of the Anderson theory of “dirty” superconductors [31].

## 5. Conclusions

In an attempt to enhance the physical-mechanical properties of the well-studied Hf-Nb-Ta-Ti-Zr refractory HEAs, we have employed the additional element scandium to produce equimolar Sc-Hf-Nb-Ta-Ti-Zr alloys. Unlike the Hf-Nb-Ta-Ti-Zr parent HEAs, which possess a single-phase bcc structure and quite homogeneous microstructure, the addition of Sc produces a two-phase structure of the Sc-Hf-Nb-Ta-Ti-Zr alloys, with one phase being bcc and the other hcp. The hcp phase absorbs practically all Sc, whereas the Sc-poor bcc phase is more or less identical to the bcc phase of the Hf-Nb-Ta-Ti-Zr parent system. Upon the addition of Sc, the microstructure becomes very inhomogeneous. Large bcc dendrites are homogeneous in the central parts, but become a fine dispersion of sub-micron precipitates of the bcc and hcp phases close to the edges. The interdendritic regions are also a similar fine dispersion of the two phases, where the hcp precipitates are in the majority. Superconductivity of the Sc-Hf-Nb-Ta-Ti-Zr alloys originates from the bcc phase fraction, which demonstrates practically identical superconducting parameters as the bcc Hf-Nb-Ta-Ti-Zr parent alloys. The superconducting phase is a BCS-like, type II superconductor. The enormous chemical and structural disorder classifies it as the Anderson “dirty” superconductor. The Sc-containing hcp phase fraction is non-superconducting.

## 6. Methods

XRD diffraction patterns were recorded on a PANalytical X’Pert PRO MPD (Malvern Panalytical Ltd., Malvern, UK) X-ray powder diffractometer in a classical Bragg-Brentano geometry, using a Ni filter that retains the Cu Kα_1,2_ doublet radiation. SEM BSE imaging and EDS composition determination and elemental mapping were performed on a focused ion beam scanning electron microscope FEI HeliosNanolab 650 (FEI, Hillsboro, OR, USA), equipped with EDS system from Oxford Instruments (Oxford, UK) and X-max SDD detector. Electrical resistivity and specific heat were measured on a Quantum Design Physical Property Measurement System PPMS 9T (San Diego, CA, USA), operating in the temperature range 0.35–400 K and equipped with a 9 T magnet. Magnetic measurements were performed on a Quantum Design Magnetic Property Measurement System MPMS3 (San Diego, CA, USA) that employs a SQUID magnetometer and operates in the temperature range 1.8–400 K in magnetic fields up to 7 T.

## Figures and Tables

**Table 1:** Properties of the elements constituting the Sc-Hf-Nb-Ta-Ti-Zr alloys (atomic radius r, melting temperature $T_{m}$, type of high-temperature and room-temperature structure, lattice parameters and the temperature of the bcc-to-hcp allotropic transition) [24,25].

Element	_21_Sc	_22_Ti	_40_Zr	_41_Nb	_72_Hf	_73_Ta
r (Å)	1.641	1.462	1.603	1.429	1.578	1.430
$T_{m}$ (K)	1812	1943	2125	2740	2500	3287
HT structure	bcc	bcc	bcc	bcc	bcc	bcc
*a* (Å)	3.752	3.320	3.616	3.305	3.615	3.365
(HT bcc)	T = 1623 K	T = 1173 K	T = 1252 K	T = 473 K	T = 2073 K	T = 2588 K
RT structure	hcp	hcp	hcp	bcc	hcp	bcc
*a* (Å)	3.313	2.951	3.242		3.196	
*c* (Å)	5.276	4.683	5.169		5.058	
(RT hcp)	T = 298 K	T = 298 K	T = 298 K		T = 299 K	
bcc-to-hcp transition (K)	1608	1150	1136		2013	

**Table 2:** Binary-mixing enthalpies (in kJ mol^−1^) for unlike atomic pairs constituting the Sc-Hf-Nb-Ta-Ti-Zr alloys [24,25].

_21_Sc	8	4	18	5	16
8	_22_Ti	0	2	0	1
4	0	_40_Zr	4	0	3
18	2	4	_41_Nb	4	0
5	0	0	4	_72_Hf	3
16	1	3	0	3	_73_Ta

**Table 3:** List of the investigated Sc-Hf-Nb-Ta-Ti-Zr alloys, their chemical composition, structure, crystallographic parameters and molar fractions of the bcc and hcp phases. The column “Appearance” describes appearance of the phase in the microstructure (in relation to the SEM BSE images). The composition of the dark phase is an average over fine dispersion of the bcc and hcp sub-micron precipitates in the interdendritic regions (with the hcp precipitates in majority), while the composition of the bright phase corresponds to the bcc dendrites.

Alloy	Appearance	Composition	Structure	Mol. Fraction (%)
#1-ScHfNbTi	bright	Sc_20_Hf_25_Nb_30_Ti_25_	bcc, a= 3.37 Å	68
	dark	Sc_33_Hf_22_Nb_22_Ti_23_	hcp, a= 3.24 Å, c= 5.12 Å	32
#2-ScHfNbZr	bright	Sc_21_Hf_26_Nb_28_Zr_25_	bcc, a= 3.47 Å	76
	dark	Sc_28_Hf_21_Nb_25_Zr_26_	hcp, a= 3.24 Å, c= 5.11 Å	24
#3-ScHfTaTi	bright	Sc_12_Hf_24_Ta_38_Ti_26_	bcc, a= 3.36 Å	66
	dark	Sc_56_Hf_20_Ta_8_Ti_16_	hcp, a= 3.23 Å, c= 5.09 Å	34
#4-ScNbTiZr	bright	Sc_18_Nb_32_Ti_24_Zr_26_	bcc, a= 3.36 Å	60
	dark	Sc_33_Nb_20_Ti_21_Zr_26_	hcp, a= 3.26 Å, c= 5.15 Å	40
#5-ScTaTiZr	bright	Sc_4_Ta_64_Ti_22_Zr_10_	bcc, a= 3.32 Å	65
	dark	Sc_32_Ta_10_Ti_26_Zr_32_	hcp, a= 3.24 Å, c= 5.11 Å	35
#6-ScHfNbTaTi	bright	Sc_8_Hf_22_Nb_25_Ta_25_Ti_20_	bcc, a= 3.37 Å	80
	dark	Sc_67_Hf_14_Nb_5_Ta_4_Ti_10_	hcp, a= 3.25 Å, c= 5.15 Å	20
#7-ScHfNbTaZr	bright	Sc_9_Hf_21_Nb_26_Ta_26_Zr_18_	bcc (L), a= 3.45 Å	75
	intermediate	Sc_15_Hf_22_Nb_22_Ta_20_Zr_21_	bcc (S), a= 3.37 Å	
	dark	Sc_36_Hf_19_Nb_12_Ta_8_Zr_25_	hcp, a= 3.25 Å, c= 5.13 Å	25
#8-ScNbTaTiZr	bright	Sc_4_Nb_29_Ta_41_Ti_15_Zr_11_	bcc, a= 3.34 Å	75
	dark	Sc_33_Nb_12_Ta_5_Ti_19_Zr_31_	hcp, a= 3.25 Å, c= 5.13 Å	25
#9-ScHfNbTaTiZr	bright	Sc_12_Hf_17_Nb_19_Ta_19_Ti_17_Zr_16_	bcc, a= 3.37 Å	77
	dark	Sc_29_Hf_15_Nb_13_Ta_12_Ti_13_Zr_18_	hcp, a= 3.24 Å, c= 5.12 Å	23

**Table 4:** Superconducting parameters of the Sc-Hf-Nb-Ta-Ti-Zr alloys, determined from (1) electrical resistivity (superconducting transition temperature $T_{C}^{\rho }$, upper critical field $H_{c2}\left(0\right)$ and the exponent β of the empirical fit to determine $H_{c2}\left(0\right)$, see text), (2) specific heat (superconducting transition temperature $T_{C}$, electronic specific heat coefficient γ, Debye temperature $\theta_{D}$ and molar superconducting fraction x of the material) and (3) magnetization (lower critical field $H_{c1}\left(0\right)$ and the exponent $\beta_{1}$ of the empirical fit to determine $H_{c1}\left(0\right)$, see text). The corresponding parameters of pure metals ($T_{C}$, γ, $\theta_{D}$) are also given (reproduced from ref. [26]).

Pure Metals									
		$T_{C}$ (K)			γ (mJ mol^−1^ K^−2^)		$\theta_{D}$ (K)		
**Sc**		/			10.34		346		
**Hf**		0.12			2.15		252		
Nb		9.20			7.80		276		
Ta		4.48			5.87		246		
Ti		0.39			3.36		420		
Zr		0.55			2.77		290		
**Sc-Hf-Nb-Ta-Ti-Zr alloys**									
Alloy	Electrical resistivity			Specific heat				Magnetization	
	$T_{C}^{\rho }$ (K)	$\mu_{0}H_{c2}\left(0\right)$ (T)	β	$T_{C}$ (K)	γ (mJmol^−1^K^−2^)	$\theta_{D}$ (K)	x (%)	$\mu_{0}H_{c1}\left(0\right)$ (T)	$\beta_{1}$
#1-ScHfNbTi	6.6	13.1	1.62	6.2	7.21	223	72	0.56	1.88
#2-ScHfNbZr	6.8	11.1	1.49	~6	5.50	213	86	0.38	1.65
#3-ScHfTaTi	6.4	14.7	1.10	6.0	6.46	220	74	0.17	2.51
#4-ScNbTiZr	8.4	13.7	1.59	7.3	7.83	259	75	1.27	1.92
#5-ScTaTiZr	7.4	13.5	1.12	6.8	7.49	231	62	0.03; 0.11	2.68; 1.37
#6-ScHfNbTaTi	6.6	13.1	1.27	6.1	7.85	214	75	0.41	2.11
#7-ScHfNbTaZr	7.4	12.4	1.01	7.2	5.88	241	61	0.23	1.49
#8-ScNbTaTiZr	7.9	17.3	1.02	7.2	8.74	254	79	0.29	2.11
#9-ScHfNbTaTiZr	7.2	14.1	1.09	6.6	6.61	219	77	0.70	1.86

## Data Availability

The data presented in this study are available on request from the corresponding authors.

## Supplementary Materials

The following supporting information can be downloaded at: https://www.mdpi.com/article/10.3390/ma15031122/s1: EDS elemental maps of the Sc-Hf-Nb-Ta-Ti-Zr refractory alloys.
