# Supplementary material for: The Effect of Scandium on the Structure, Microstructure and Superconductivity of Equimolar Sc-Hf-Nb-Ta-Ti-Zr Refractory High-Entropy Alloys
Source: Materials (Basel). 2022 Jan 31;15(3):1122. doi: 10.3390/ma15031122 (PMC8838456; doi:10.3390/ma15031122)

Supplementary Materials

The Effect of Scandium on the Structure, Microstructure and Superconductivity of Equimolar Sc-Hf-Nb-Ta-Ti-Zr Refractory High-Entropy Alloys

Mitja Krnel [1], Andreja Jelen [1], Stanislav Vrtnik [1], Jože Luzar [1], Darja Gačnik [1], Primož Koželj [1,2], Magdalena Wencka [1,3,] Anton Meden [4], Qiang Hu [5,*], Sheng Guo [6] and Janez Dolinšek [1,2,*]

1 Jožef Stefan Institute, Jamova 39, SI-1000 Ljubljana, Slovenia; mitja.krnel@ijs.si (M.K.); andreja.jelen@ijs.si (A.J.); stane.vrtnik@ijs.si (S.V.); joze.luzar@ijs.si (J.L.); darja.gacnik@ijs.si (D.G.); primoz.kozelj@ijs.si (P.K.); magdalena.wencka@ijs.si (M.W.)
2 Faculty of Mathematics and Physics, University of Ljubljana, Jadranska 19, SI-1000 Ljubljana, Slovenia
3 Institute of Molecular Physics, Polish Academy of Sciences, Smoluchowskiego 17, PL-60-179 Poznań, Poland
4 Faculty of Chemistry and Chemical Technology, University of Ljubljana, Večna pot 113, SI-1000 Ljubljana, Slovenia; anton.meden@fkkt.uni-lj.si
5 Institute of Applied Physics, Jiangxi Academy of Sciences, Changdong Road 7777, Nanchang 330096, China
6 Industrial and Materials Science, Chalmers University of Technology, SE-41296 Göteborg, Sweden; sheng.guo@chalmers.se
* Correspondence: huq@jxas.ac.cn (Q.H.); jani.dolinsek@ijs.si (J.D.)

EDS elemental maps of the investigated Sc-Hf-Nb-Ta-Ti-Zr refractory alloys:

a) #1-ScHfNbTi

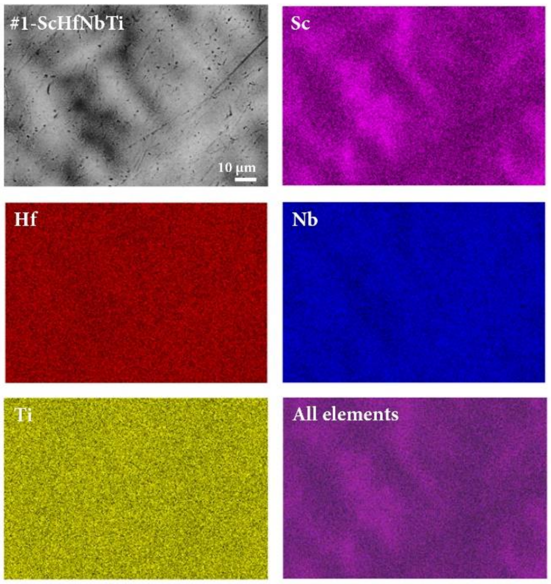

b) #2-ScHfNbZr

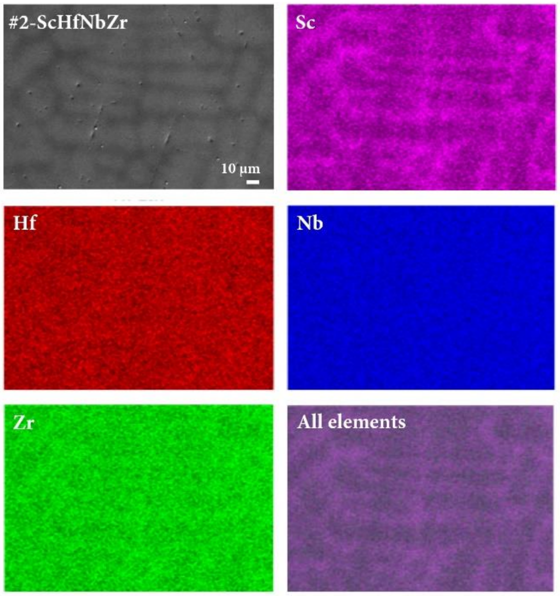

c) #3-ScHfTaTi

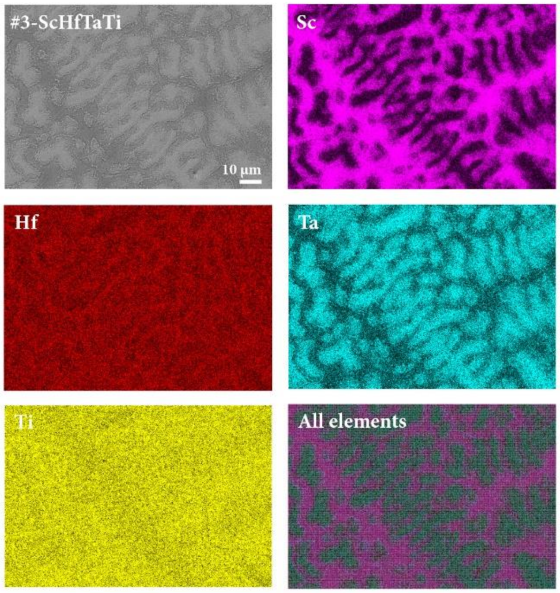

d) #4-ScNbTiZr

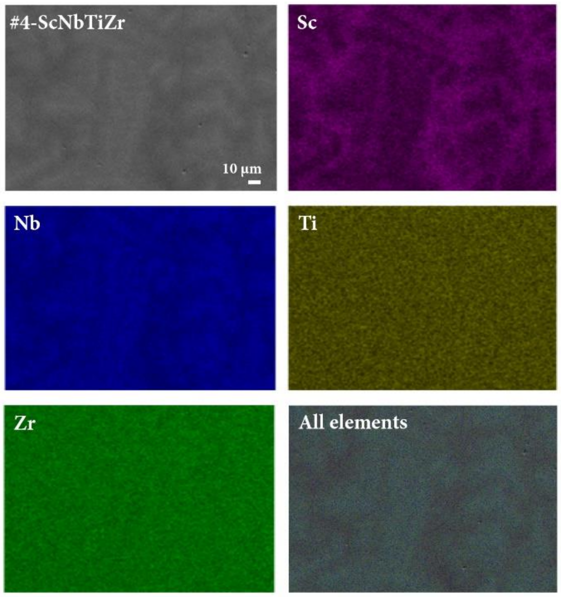

e) #6-ScHfNbTaTi

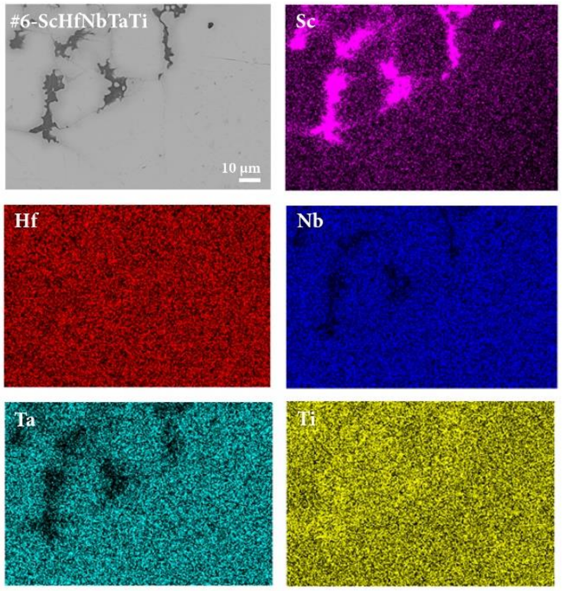

f) #7-ScHfNbTaZr

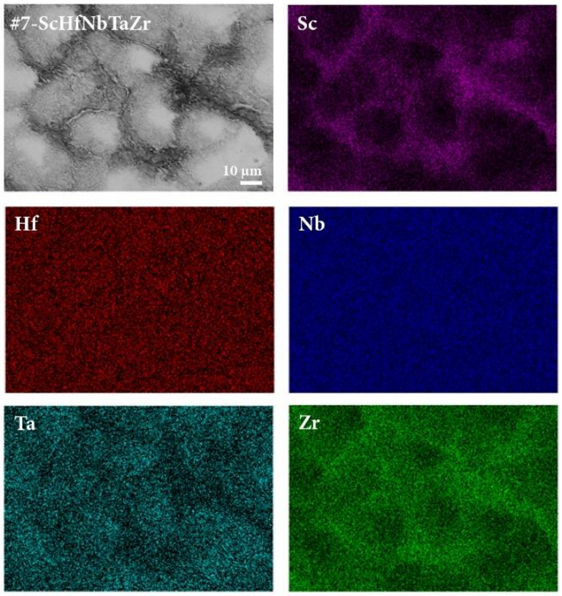

g) #8-ScNbTaTiZr

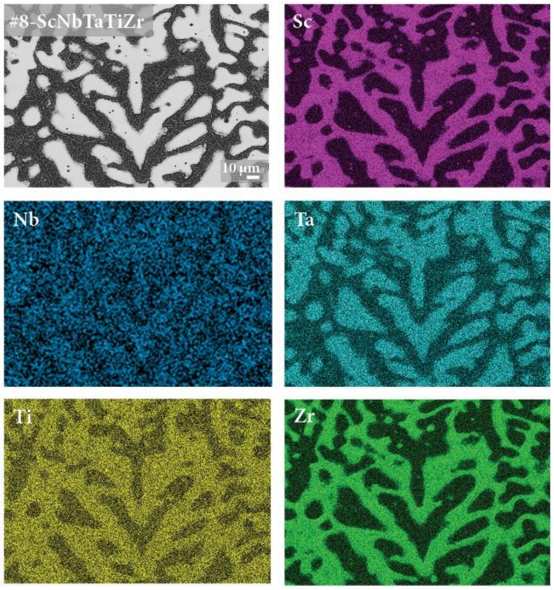

h) #9-ScHfNbTaTiZr

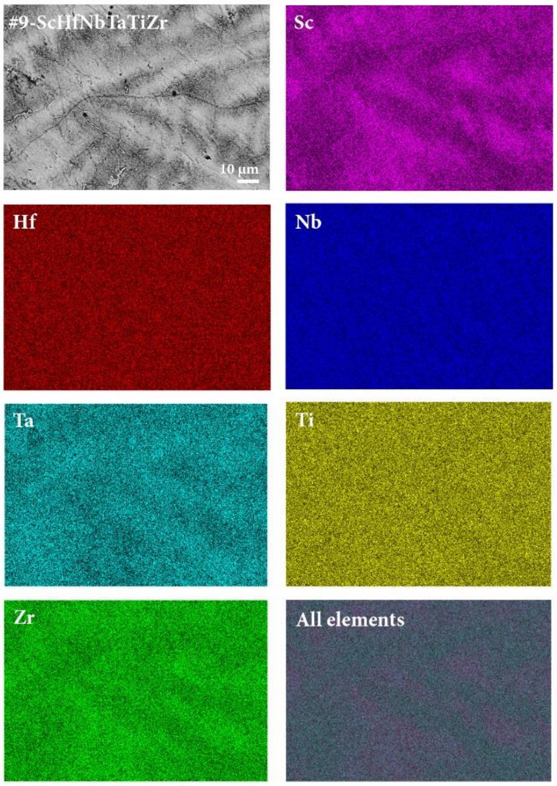

Supplement: Supplementary file 1 [file materials-15-01122-s001.zip › materials-1547922-supplementary.pdf]
